# Predictors of long-term abstinence in a randomized controlled trial of smoking cessation by mobile phone text messaging (‘Happy Quit’) in China

**DOI:** 10.18332/tpc/152255

**Published:** 2022-08-05

**Authors:** Yanhui Liao, Yunfei Wang, Jinsong Tang, Qiuxia Wu, Zhenzhen Wu, Ann McNeill

**Affiliations:** 1 Department of Psychiatry, Sir Run Run Shaw Hospital, Zhejiang University School of Medicine, Hangzhou, Zhejiang, China; 2 Addictions Department, Institute of Psychiatry, Psychology and Neuroscience, King’s College London, London, United Kingdom; 3 Department of Psychiatry, and National Clinical Research Center for Mental Disorders, the Second Xiangya Hospital of Central South University, China; 4 Ningbo Kangning Hospital, Zhejiang Province, Ningbo, China

**Keywords:** long-term abstinence, predictors, text messaging-based, ‘Happy Quit’, smoking cessation, randomized controlled trial

## Abstract

**INTRODUCTION:**

The mobile phone-based text messaging intervention (‘Happy Quit’) is a minimal and effective intervention with very wide reach; thus, it has the possibility of a population impact on quitting rates. Obtaining information on predictors of long-term quit rates is crucial for developing and implementing more effective mobile-based interventions. The study aimed to explore the predictors of long-term abstinence following the ‘Happy Quit’ intervention.

**METHODS:**

This study is a secondary analysis of a randomized controlled trial (RCT) that compared 12-week text messaging intervention (‘Happy Quit’) versus control intervention with follow-up at 24 weeks, in China. Only participants who had biochemically verified continuous smoking abstinence at 24 weeks were followed up at 52 weeks after the quit date. This predictor regression analysis is for those who were biochemically verified continuous 52-week quitters (n=67) compared with the other participants (n=1302) in the RCT.

**RESULTS:**

Of the 69 smokers who were continuously abstinent at 24 weeks, 97.1% (n=67) remained continuously abstinent at 52 weeks. The biochemically verified long-term (52 weeks or 1year) quit rate was 6.3% in the intervention group (60/958), 1.7% in the control group (7/411) (OR=3.677; 95% CI: 1.67–8.11, p<0.001). Multivariable regression analysis revealed that only smoked ≤10 cigarettes per day (compared with >10 cigarettes per day) was the only predictor for long-term abstinence.

**CONCLUSIONS:**

This study suggests that individuals who are light smokers might get the most benefit from the text messaging intervention (‘Happy Quit’) in China.

## INTRODUCTION

China had over 300 million (overall: 26.6%, male: 50.5%, and female: 2.1%) current smokers in 2018^1^. The ‘Healthy China 2030 Strategy’ set a goal to reduce adult smoking prevalence to 20% by 2030^2^. Boosting quit rates is considered to be the most important and only strategy that can determine a significant decrease in smoking-related disability and premature death in the short-term. ‘Happy Quit’^3^ is a text messaging smoking cessation intervention that has proven effective in promoting smoking cessation.

Obtaining information on predictors of long-term successful cessation is crucial for developing and implementing more effective interventions via digital devices at the individual and population level. Research by Horn et al.^4^ revealed that light smokers were more likely to maintain long-term abstinence than heavy smokers in adolescents. However, little is known about which subgroups of adults could get more benefit from the digital intervention of ‘Happy Quit’.

This study aimed to explore the association of demographic and smoking indicators with long-term cessation (biochemically verified quit for at least one year) among adult participants from the ‘Happy Quit’ study^3^. Furthermore, the study tests the hypothesis that light smokers have more chance to sustain long-term abstinence than their heavy smoking counterparts.

## METHODS

### Participants and settings

This trial assessed 2561 participants between 17 August 2016 and 27 May 2017. A total of 1369 participants [including 674 participants from a high-frequency messaging (HFM, 3 to 5 messages/day) group, 284 participants from a low-frequency messaging (LFM, 3 to 5 messages/week) group, and 411 participants from a control group (1 non-interventional message/week)] from a single-blind, randomized controlled trial (RCT) of a text-messaging-based smoking cessation intervention (‘Happy Quit’) were included in this analysis. The text message library of ‘Happy Quit’ in Chinese (Supplementary file). The trial was registered at ClinicalTrials.gov (Identifier: NCT02693626). A detailed description of participants can be found in the published protocol^5^ and results of abstinence rates at 24 weeks follow-up in the outcomes study^3^. The inclusion criteria were: daily cigarette smokers; ≥18 years of age; currently living in China; being able to read and write in Chinese; owning a text-capable cell phone and knowing how to text; willing to make an attempt to quit smoking in the next month; and willing to provide informed consent. This text messaging-based intervention had no restrictions on setting or location.

A total of 69 participants among 1369 participants with biochemically verified continuous smoking abstinence at 24 weeks were followed to 52 weeks (one year) after the quit date, including 61 participants from the intervention groups (44 in the HFM group, and 17 in the LFM group) and 8 participants from the control group. A total of 67 participants with self-reported continuous smoking abstinence at 52 weeks provided biochemical verification.

The study was conducted in accordance with the Declaration of Helsinki. This study protocol was approved by the Second Xiangya Hospital of Central South University Review Board [No. S007 (2015) and No. S111 for adding intervention and follow-up (2016)]. Agreement of informed consent form by text message was obtained from each participant. Participants were fully informed about the purpose, procedures, measurements, and potential risks and benefits of the study. All personal information had been de-identified. For more detailed ethics approval and consent see the study protocol^5^. The 52-week (one-year) follow-up was also approved by the same review board [No. S111 (2015)]. No technical problems or other unintended incidents were reported.

### Outcome and outcome measures

The primary outcome for this analysis was biochemically measured long-term abstinence (52 weeks, see below). The secondary outcomes were the predictor variables for long-term abstinence using baseline data from ‘Happy Quit’, which included age, gender, level of education, number of previous quit attempts and number of cigarettes smoked per day (from Fagerström test of nicotine dependence, FTND).

Long-term abstinence/quit in this study was defined as biochemically verified continuous smoking abstinence at 52 weeks. This study used a strict criterion for smoking abstinence: ‘not even a single puff of smoke’ between the quit day and follow-up at 52 weeks. A total of 67 participants with self-reported 52-week continuous smoking abstinence received cotinine (nicotine metabolite) urine dipsticks with detailed instructions and 20 Chinese Renminbi (about 2.96 US$) in cash by mail. This study applied a urine cotinine cut-off point of 200 ng/mL, and all test results were digitally sent back to research assistants.

### Statistical analysis

Intention-to-treat (ITT) analysis was applied to determine smoking abstinence rates. A total of 1369 participants were all included in the analysis. As the sample for identifying long-term quitters (biochemically verified continuous smoking abstinence at 52 weeks) was small, this study combined long-term quitters from both groups. Rates of long-term quitters were calculated as percentages. Descriptive and unadjusted univariate analysis were used for demographic characteristics, smoking behavior at baseline among overall sample, long-term quitters and continuing smokers. Multiple risk factors, including gender, age (<30, ≥30), previous quit attempts (never, one or more attempts), and cigarettes per day (≤10, >10), were explored by logistic regression analysis (LRA). All statistical analyses were performed using SPSS version 22. A two-sided p<0.05 was used to determine statistical significance.

## RESULTS

This study only followed those who had biochemically verified continuous smoking abstinence at 24 weeks (n=69), which occurred in 61/958 participants in the intervention group (6.4%), and 8/411 participants (1.9%) in the control group (p=0.004). From the follow-up at 24 weeks to follow-up at 52 weeks after the quit date, 1 participant from the control group lost contact and 1 participant from HFM group reported relapse among 69 biochemically verified 24-week quitters, but the remaining 67 participants (97.1%) were biochemically verified abstainers. Thus, the biochemically verified continuous smoking abstinence at 52 weeks was 6.3% in the intervention group (60/958), and 1.7% in the control group (7/411) (OR=3.677; 95% CI: 1.67–8.11, p<0.001).

Table 1 shows the baseline characteristics of overall participants, quitters, smokers and long-term quit rates. Long-term quit rates were relatively high among those participants who were aged ≥30 years, smoked ≤10 cigarettes per day, or had made previous quit attempts prior to this study. The long-term quit rate was 10.26% (4/39) in participants who were aged >60 years and 7.14% (3/42) in participants who had ≥6 quit attempts.

**Table 1:** Baseline characteristics of all participants, quitters, smokers

	*All participants (N=1369) mean ± SD or n (%)*	*Long-term quitters (n=67) mean ± SD or n (%)*	*Smokers (n=1302) mean ± SD or n (%)*	*Quit rate^#^*	p*
**Age** (years)	38.1 ± 9.79	40.4 ± 9.91	38.0 ± 9.77		0.044
<30	287 (21.0)	8 (11.9)	279 (21.4)	2.79	
<25	80 (5.8)	1 (1.5)	79 (6.1)	1.25	
25–29	207 (15.1)	7 (10.4)	200 (15.4)	3.38	
≥30	1082 (79.0)	59 (88.1)	1023 (78.6)	5.45	
30–39	521 (38.1)	28 (41.8)	493 (37.9)	5.37	
40–49	372 (27.2)	18 (26.9)	354 (27.2)	4.84	
50–59	150 (11.0)	9 (13.4)	141 (10.8)	6.0	
≥60	39 (2.8)	4 (6.0)	35 (2.7)	10.26	
**Gender**					0.834
Male	1295 (94.6)	63 (94.0)	1232 (94.6)	4.86	
Female	74 (5.4)	4 (6.0)	70 (5.4)	5.71	
**Education level** (years)					0.792
≤12	349 (25.5)	18 (26.9)	331 (25.4)	5.16	
>12	1020 (74.5)	49 (73.1)	971 (74.6)	4.80	
**Number of cigarettes smoked per day**	20.1 ± 9.19	18.9 ± 9.99	20.2 ± 9.15		0.279
≤10	244 (17.8)	20 (29.9)	224 (17.2)	8.20	
>10	1125 (82.2)	47 (70.1)	1078 (82.8)	4.18	
11–20	799 (58.4)	32 (47.8)	767 (58.9)	4.01	
21–30	220 (16.1)	9 (13.4)	211 (16.2)	4.09	
≥30	106 (7.7)	6 (9.0)	100 (7.7)	5.66	
**Previous quit attempts**	1.5 ± 4.59	1.8 ± 4.79	1.5 ± 4.58		0.550
0	583 (42.6)	23 (34.3)	560 (43.0)	3.97	
≥1	786 (57.4)	44 (65.7)	742 (57.0)	5.60	
1–5	744 (54.3)	41 (61.2)	703 (54.0)	5.51	
≥6	42 (3.1)	3 (4.5)	39 (3.0)	7.14	
**FTND score**	4.6 ± 2.16	4.1 ± 2.55	4.6 ± 2.14		0.053
<4	418 (30.5)	24 (35.8)	394 (30.3)	5.74	
≥4	951 (69.5)	43 (64.2)	908 (69.7)	4.52	

# Quit rates: (quitter/all participants)×100; the overall quit rate=(67/1369)×100=4.89%. Quitters: participants who had achieved biochemically verified continuous smoking abstinence at 52 weeks. Smokers: participants other than those who had not achieved biochemically verified continuous smoking abstinence at 52 weeks. FTND: Fagerström test for nicotine dependence; FTCD score <4: minimal dependence; FTCD score ≥4: moderate to high dependence.

* Significant difference, p<0.05 (univariate analysis, unadjusted).

Variables predicting long-term abstinence: the multivariable LRA indicated that only smoked ≤10 cigarettes per day (compared with >10 cigarettes per day) was the only predictor for long-term abstinence (OR=0.43; 95% CI: 0.25–0.76, p=0.003).

## DISCUSSION

This analysis indicates that light smoking (smoked ≤10 cigarettes per day) was a significant predictor of long-term abstinence in a trial of a text messaging-based smoking cessation intervention, and that text messaging interventions may improve long-term smoking cessation rates as well as short-term rates.

The results showed that, with 12-week interventions after quit date, the biochemically verified continuous smoking abstinence at 52 weeks was 6.3% in the intervention group (60/958), and 1.7% in the control group (7/411), which were similar to the results of the follow-up at 24 weeks (6.4% in the intervention group, and 1.9% in the control group)^3^. This study suggests that if the participants can maintain abstinence (not even one puff of smoke) for a period of 24 weeks, they are very likely to maintain long-term abstinence for a period of 52 weeks (one year). This finding is consistent with analysis of shape of the relapse curve among untreated smokers, which indicates that most relapse occurs in the first 2 weeks; the curve becomes relatively sustainable after 3 months^6^. Similarly, by analyzing data from the *txt2stop* trial of sending a lapse text requesting support, half of all lapse texts arrived within the first 1–3 weeks after the quit date^7^.

However, the long-term quit rates in ‘Happy Quit’ are lower than those of most other studies^8,9^. Shortage of smoking cessation services (even within hospitals)^10^, and widespread of cigarette gifting and sharing in China, may greatly discourage smoking cessation and contribute to the low quit rates.

This study found that smoked less cigarettes per day was a significant predictor of long-term abstinence. Those smokers might derive major benefit from effective smoking cessation interventions. First line pharmacotherapy such as nicotine replacement therapy (NRT), bupropion, and varenicline, should also be recommended, especially to heavy smokers. Not all participants can evenly gain benefit from ‘Happy Quit’. Thus, it is important to develop and disseminate a more effective smoking cessation program for subgroups such as young adults and heavier smokers.

### Strengths and limitations

A strength of this study is that self-reported long-term (at both 24 weeks and 52 weeks) abstinence had been confirmed by biochemical verification (urine cotinine verified abstinence).

This study has some limitations. First, although this is the first text messaging-based large sample size RCT for smoking cessation in China, we only followed a very small group of people (n=69) who reported continuous smoking abstinence at 24 weeks and confirmed by urine cotinine test. Second, as the sample size for long-term quitters was small and only 7 participants from the control group, this analysis combined the long-term quitters from both intervention and control groups. The predictors for long-term abstinence in the control group might be different from the intervention group. It is worthwhile to explore these groups of individuals separately with larger sample size in the future. Finally, some other factors, such as motivation and self-efficacy, should also be explored in the future.

## CONCLUSIONS

This study demonstrated that individuals who are light smokers might get the most benefit from mobile phone-based text messaging intervention (‘Happy Quit’). It also demonstrated the long-term (52 weeks) efficacy of ‘Happy Quit’ for smoking cessation in China. It is worth pointing that the ‘Happy Quit’ is a minimal intervention with very wide reach, thus it has the possibility of a population impact on quitting rates.

## Data Availability

The data supporting this research are available from the authors on reasonable request and with completion of a data user agreement.
