# Supplementary material for: Predictors of long-term abstinence in a randomized controlled trial of smoking cessation by mobile phone text messaging (‘Happy Quit’) in China
Source: Tob Prev Cessat. 2022 Aug 5;8:31. doi: 10.18332/tpc/152255 (PMC9354670; doi:10.18332/tpc/152255)
Supplement: Supplementary file 1 [file TPC-8-31-s1.pdf]

Text Message Library of “Happy Quit” in Chinese
附件：“快乐戒烟”短信库

戒烟前的准备工作

欢迎信

欢迎参加“快乐戒烟”活动， 您还可以随时通过其他方式（具体提供的免费戒烟服务如电话服务、邮件等），进行免费咨询。回复“退出”，可以随时退出此活动。

基本信息（**※说明：**可以根据具体研究或临床应用修改）

1. 性别（1 男，2 女）
2. 年龄（周岁）
3. 学历（1 小学，2 初中，3 高中，4 大学，5 研究生及以上）
4. 有过几次戒烟经历（__次）

尼古丁依赖测量问卷(FTCD) 评分及分析

请选择一个最适合您的选项，并打“√”。

题目	3 分	2 分	1 分	0 分
1. 一般来说，您早晨醒来后多长时间吸第一支烟	〈=5 分钟	5-30 分钟	31-60 分钟	〉60 分钟
2. 早晨醒来后第一个小时内的吸烟是否多于其它时间?			是	否
3. 最不愿意放弃哪种情况下的吸烟?			早上第一支	其他情况
4. 您在禁止吸烟的场所如图书馆、电影院时是否会感到不吸烟难以坚持？			是	否
5. 您是否在患病卧床期间仍在吸烟?			是	否
6. 您每天吸多少支烟？	〉=30 支	21-30 支	11-20 支	〈=10 支

评分标准：

<4 分：尼古丁轻度依赖（还好您对尼古丁的依赖程度不高，现在就下定决心，一定可以戒烟成功！）

4-6 分：尼古丁中度依赖（您对尼古丁的依赖程度偏高，所以要好好加油、努力戒除烟瘾！）

7-10 分：尼古丁重度依赖（您对尼古丁的依赖程度非常的高！建议您同时使用戒烟药物！）

准备一个日记本

请您为自己准备一个小小的日记本，写下您的戒烟原因，记录您的戒烟过程，包括戒烟第几天，每天吸烟数量，什么情况最容易吸烟，每天的感受，身体的变化等。我们也将每周了解您的戒烟情况。如果您不想另外准备日记本，您也可以利用这本手册的附录 5 进行记录。（建议戒烟前的准备工作时间为 1 周-2 周左右）

戒烟前的干预（2 周左右）

※说明：*“快乐戒烟”项目发送短信的时间主要是在早上 7：30，中午 12：30 和晚上 7：30 左右。可以调整对特定地区或人群发送短信的时间。*

- 我们每天将发送 1 到 3 条短信给您，您在任何时候都可以获得帮助，回复数字：
    - “7”（如何应对吸烟“渴求”）
    - “8”（如何调整“心情”）
    - “9”（“为什么戒烟”）
- 您还可以随时通过（具体方式），进行免费咨询。

※ **说明：**以上内容可以每周重复。可以参考下面列出的针对“应对渴求”、“调整心情”和“为什么戒烟”的回复内容，回复内容会随机出现。部分短信内容也可以进行重复。

- 想想有什么能让您开心的，听音乐？跑步？游泳？逛街？做任何能让您开心的事情，但是别吸烟。明天您将感觉更高兴。
- 戒烟挑战时间到了。请试着在 4 小时内不吸烟。当有吸烟渴求时做深呼吸（放松，慢慢的深吸气，慢慢的呼气）。
- 问问自己在过去 4 小时内有没有吸烟，并记录下来。如果您挑战成功，恭喜您！尝试挑战更长的时间不吸烟，这将有利于您成功戒烟；如果您挑战失败也没关系，再次尝试，直到挑战成功。
- 戒烟是一个艰难的任务，试着多吃蔬菜水果、多喝水、保持充足的睡眠，让自己更健壮。
- 戒烟前，请记录您每天吸多少支烟，试着每天减少 1/4，如果您每天吸烟 20 支，尝试减少为 15 支。我们稍后会对您今天的吸烟情况进行检查。
- 阻止吸烟继续危害您健康的唯一方法是戒烟。
- 请在日记本上写下您昨天吸了多少支烟，问问自己是否减少了 1/4，如果达到目标，恭喜您！如果没有也不要放弃哦，加油！
- 还有 3 天就是您的戒烟日了，想一想您在什么情况下最想吸烟？压力过大？聚餐？尝试改变您的吸烟模式，尽量避免参加让您想吸烟的活动。
- 我相信最艰难的行为就是选择戒烟，您已经做到了！还有 3 天的准备时间，相信自己一定能戒烟！
- 记住每天第一件事情就是记录您昨天的吸烟情况，问问自己有没有达到减少 1/4 的目标。
- 您想戒烟吗？和烟、打火机、火柴及烟灰缸说再见吧！从您的家中、车上、办公室里，把所有和吸烟有关的物品清除干净。
- “我戒烟，是因为我不想将自己辛苦挣的钱浪费在买烟上。”
- 别忘记记录您昨天的吸烟情况。您的戒烟日马上就要到了，您是否为更健康长寿的生活做好准备了？相信您一定准备好了！请在您的笔记本写下“我已经为彻底戒烟准备好啦！”

- 您抽的每一支烟都会危害您和家人的健康。还有两天就是您的戒烟日了！
- 现在请为戒烟做准备，想一想如果有人递烟给您的时候您应该怎么说？尝试练习说："不，谢谢，我不吸烟了"。
- 明天将是一个非常重要的日子！请再次确认吸烟相关物品（如香烟、打火机、烟灰缸）是否已经清除，从戒烟日开始就一支烟也不抽啦，从此您将成为不吸烟者。
- 明天是一个重要的日子，请一定清除吸烟相关物品（特别是烟、打火机和烟灰缸）。
- 再想想有没有吸烟相关物品没有清理的？让自己忙碌起来，避免到以前常吸烟的地方，避免和吸烟的朋友在一起。看着镜子，告诉自己：明天将开始过没有烟草危害的日子，相信自己一定能做到！

戒烟日的干预

- **重要的日子：**今天就是您的戒烟日！戒烟，您确定准备好了吗？请大声说出"我已经准备好了！"您一定能成功！不要放弃！请告诉您身边所有的人，获得他们的支持。
- **心理准备：**当您想吸烟的时候，试图做深呼吸、多喝水、吃点零食、做点别的事情让自己忙碌起来，告诉自己等会儿吸烟的渴求就会慢慢减少或消失。
- **行为准备：**运动起来，因为运动可以释放压力，降低吸烟渴求。不要熬夜，保持充足的睡眠。切记清除所有的香烟、打火机、烟灰缸等与吸烟相关的物品。

戒烟后的干预（1-12 周）

第一周（示例每天发送的短信内容）

戒烟第 1 天

1. 做的非常好！恭喜您迈出了重要的一步！我们的建议是：常常练习说"不，谢谢，我现在不吸烟了！"
2. 请做好准备，您在戒烟后可能会感觉不适，这是正常的现象，是因为您对尼古丁产生了依赖，所以戒烟后会出现一系列的戒断症状，这些不适感会随时间的延长而逐渐消失。
3. 问问自己今天是否完全没有吸烟？如果完全没有吸烟，我们为您感到高兴，做得非常好！如果吸烟了，也不要放弃，我们知道戒烟非常艰难，有时需要很多次尝试才能成功，争取明天开始完全不吸烟。

戒烟第 2 天

1. 当您又想吸烟时，请坚持不吸或试着忍耐 5 分钟。请开始努力戒烟吧，加油！
2. 我们很高兴您坚持戒烟 2 天了，如果您忍不住吸了烟，记录您是在什么情况下吸烟的。别忘了，戒烟才是正确的道路。
3. 检查一下自己今天是否吸烟，并记录下来。相信您一定能在彻底戒烟的道路上获得成功，别放弃！

戒烟第 3 天

1. 您昨天是否一整天都没有吸烟？如果是，非常了不起！记得为自己庆祝一下，买点好吃的，庆祝自己的成功。如果吸烟了，想想是在什么情况下吸烟的，为以后避免在此情况下吸烟做准备。
2. 有没有其他方式可以缓解戒烟的不适呢？您可以尝试慢跑、散步、嚼口香糖、嗑瓜子等方法。
3. 每位戒烟者都会出现特别想吸烟的冲动，这种渴求不会马上消失，但是坚定的戒烟信念可以减弱渴求，记录一下您现在的渴求程度：高，中，低？记住，再大的吸烟冲动也会在几分钟后慢慢减少并消失，集中注意力做别的事情，时刻提醒自己是不吸烟者。

戒烟第 4 天

1. 想一想您戒烟的原因，并记录下来，每天大声朗读，特别是在您心情低沉的时候。戒烟的最初几天是最艰难的。
2. 停止吸烟有数不清的好处，如气色变好、口气清新、胃口改善、精力充沛等。您最期待其中的哪一项呢？和朋友聊聊吧。
3. 心情低落可能会导致吸烟，如果您觉得心情不好，告诉自己这只是短暂的，大多数戒烟者都会出现这种现象。告诉您的家人朋友(如果您不说或许他们不知道)，获得他们的帮助和支持。

戒烟第 5 天

1. 为什么要戒烟呢？请您再次写出理由吧！把它放在您每天都能看到的地方，以便随时提醒您、帮助您戒烟。
2. 5 天没有吸烟啦！吸烟会损害您的味觉，幸运的是，您的味觉在您戒烟几天后就能恢复。犒劳一下自己，不妨吃顿好吃的，但是避免邀请吸烟者参加。
3. 尽量远离可能引起您吸烟的人，避免二手烟。这样您会发现更容易应对吸烟渴求，等待 5 分钟渴求将慢慢消失，同时让您的嘴忙碌起来，可以嗑瓜子、嚼口香糖等。

戒烟第 6 天

1. 有什么原因使您想吸烟？人、事情、东西还是地点？列出来并找到相应的对抗办法。现在就开始吧，让您的戒烟道路更通畅。
2. 您可能忍不住吸了一口或一支烟，千万别为自己找“就吸了一口”这样的借口，记住赶紧丢掉手中的烟，清除吸烟相关的所有物品，马上行动！请记住，吸烟的渴求会随着戒烟时间延长而降低。如果您完全没有吸烟，非常棒！5 天没有吸烟了，恭喜您！
3. 千万不要因为不开心的事情而吸烟，戒烟的旅途虽然艰辛，但是非常值得！试图放松自己，比如听听音乐、散散步、聊聊天或洗个热水澡。

戒烟第 7 天（第一周，检查日）

※说明：根据情况可以每周或更长时间检查吸烟状态，此内容后面不再重复。

1. 检查时间到了：过去的 7 天里您吸烟了吗？如果您一口也没吸请回复“0”，否则请回复“1”。

i. 如果回复“0”：您一周没有吸烟！赶紧记录下来！算一下您节省了多少钱。今天一定要

为自己庆祝一下。

ii. 如果回复“1”：请告知您过去一周平均每天吸烟多少支？例如 5 支，回复：5。这种情况，请不要气馁，因为大多数戒烟成功者都经历过很多次失败。建议您回到戒烟前的准备阶段，充分做好再戒烟的准备工作，从戒烟日开始不再吸烟；您也可以从现在开始不再吸烟（一口都不吸），继续戒烟进程。

2. 当觉得想吸烟的时候，不妨去散步，散步可以帮助您控制体重，释放压力，让您觉得更有活力，或者看场电影（请记住：到不允许吸烟的电影院）。
3. 戒烟后，由于吸烟造成的健康损害就会得到逐步的缓解。我相信您现在一定呼吸畅快，咳嗽减少。记住练习深呼吸、多喝水、做点别的事，特别想吸烟时告诉自己等 5 分钟，吸烟渴求将慢慢消失。

戒烟干预（2-12 周）

※说明：可根据具体情况调整短信内容的频率。短信内容可以适当重复。

- 戒烟越早，对健康的益处越大。戒比不戒好，早戒比晚戒好。
- 不要让烟瘾挡住您戒烟的脚步。做一些运动，例如慢跑、散步、跳舞或者打太极拳等，都可以帮助您更快地克服烟瘾。
- 烟瘾会随时间的延长而减轻。用其它事情转移注意力 10 分钟，看看效果。如果还想吸烟，请再试 10 分钟，您离戒烟成功又近了一步!
- 您每年花多少钱买烟？设想一下，戒烟可以省下多少钱？您可以用这些钱做哪些更有意义的事情？
- 努力戒烟吧，您会成为孩子的好榜样。
- 放松自己，试着听听音乐、散散步或者洗个热水澡。
- 找出支持您戒烟的人，如朋友、家人或同事，在烟瘾难耐时您可以跟他们聊聊来获得支持。
- 您吸烟也会导致您家人患上多种疾病，在吸烟前请考虑家人的健康。
- 您准备越充分，戒烟就越顺利。把生活安排紧凑，让自己忙起来，您就不会想吸烟了。
- 深呼吸！困难是暂时的，烟瘾也是暂时的。多和支持您戒烟的人在一起，这将对您很有帮助。
- 在戒烟过程中感觉烦躁或脾气暴躁，这都是正常的表现，但是这些是能够完全克服的，相信自己!
- 吸烟成瘾是一种慢性疾病，这使戒烟变得很难。坚持不住的时候，记得去戒烟门诊找医生。
- 为了舒畅地呼吸、清新的口气、充满活力的容颜，请坚持戒烟吧!
- 戒烟过程中多吃水果或蔬菜，进行体育锻炼，这将帮助您减轻压力，增加信心，也让您的生活更加美好。

- 今天压力很大？千万不要依靠吸烟来缓解压力。出去活动活动、听听音乐、喝点水或者吃点东西，或许这些方法更有益。
- 在戒烟的过程中，吸烟是很大的诱惑，但是您需要知道如果半途而废，您的家人会感到十分失望的。
- 吸烟成瘾是一种疾病，因此戒烟不是那么的简单。
- 当您觉得难以控制自己想吸烟的时候，记住您一定可以做到不吸烟！
- 您知道吗？吸烟还可导致骨密度下降并影响骨折的愈合。
- 在戒烟期间每天喝大量的水，既可以保证您体内水分充足，也可以帮您抵抗烟瘾。
- 让我们开始吧！薄荷糖、水和零食可以帮您克服一天的烟瘾。生活就是由选择构成的，戒烟是您做出的正确选择。
- 尽量避开那些让您想到吸烟的人、事物和地点。真正的好朋友会理解并为您着想的。
- 烟瘾再强烈，也会逐渐减轻。转移您的注意力并时刻提醒自己为什么戒烟。
- 试试无糖口香糖、薄荷糖、牙签、吸管或瓜子，不要让您的嘴巴闲下来。
- 继续保持戒烟！需要额外的支持吗？您可以拨打全国戒烟专线：400-888-5531，或到戒烟门诊寻求帮助。
- 我们知道戒烟十分困难，但请坚持住！您一定可以成功！做一些能让您心情愉悦的事吧，但是请不要吸烟。
- 在戒烟的过程中，一个人的力量可能是不够的。建立一个支持您戒烟的朋友圈吧。
- 将口香糖、薄荷糖放在口袋里，如果有人给您递烟，您可以将口香糖、薄荷糖递给他。这是一个礼貌拒烟的好办法呦！
- 戒烟 1 个多月啦！吃东西也更香了吧？吸烟会让味蕾功能减弱，但幸运的是戒烟后 48 小时内，您的味蕾功能就会恢复正常。
- 烟瘾会随时间的延长而减轻。用其它事情转移注意力 10 分钟，看看效果。如果还想吸烟，请再试 10 分钟，您离戒烟成功又近了一步!
- 远离吸烟伤害的唯一办法就是彻底戒烟，而不是减少吸烟量。
- 我们知道有时候戒烟十分困难，但是请坚持住！请记住，无烟的生活更健康。
- 戒烟很困难，您会遇到很多阻碍。但是不要放弃，马上就要成功了！千万别拿个别吸烟者长寿的例子作为自己吸烟的借口。世界卫生组织提示，每两位吸烟者中会有一位死于吸烟相关疾病。您若吸烟，想想您将会是这两位中的哪一位？
- 想吸烟的时候，想想您戒烟的原因，以及通过戒烟所得到的好处吧。
- 您可能不像刚开始戒烟时那么自信了，也许还会考虑放弃。不管怎样都要坚持住。为了健康，戒烟是能做到的！
- 总是在同一时间段想吸烟吗？总是和同一个人在一起时想吸烟吗？了解您吸烟的诱发因素是克服烟瘾的重要环节。
- 休息一下让自己放松。小睡一会儿、洗个澡、跑步或者大喊几声，这些活动可以帮您克服烟瘾。您一定可以成功！

- 吃零食可以帮助您克服烟瘾，但要注意选择，少吃些高热量食品，小心发胖喔！
- 如果您的朋友或家人也吸烟，请他们不要在您周围吸烟，或者请他们将烟放置在您看不到的地方。
- 如果您坚持不吸烟，烟瘾会一天天减轻。
- 烟瘾再强烈，也会逐渐减轻。转移您的注意力并时刻提醒自己为什么戒烟。
- 锻炼可以帮您减轻压力，从而降低您对吸烟的渴求。请每天抽出一点时间来锻炼身体吧！
- 戒烟后的生活很美好，学会享受无烟的生活吧！
- 需要做件事情帮您转移注意力吗？约上朋友聊聊天或看看电影、享受点水果和小零食吧!
- 随着时间的流逝，烟瘾也会减弱。
- 在车内吸烟是十分危险的，即使打开车窗，也不能避免二手烟雾的危害！
- 烟不会使您的聚会更精彩，在有人向您递烟时要学会拒绝！可以带点口香糖，别人递烟时您可以送对方一块口香糖来拒绝。
- 您在业余时间会做点什么呢？在没有烟的环境里，读一本心爱的书或杂志，听一张新专辑或做一道新菜，是不是很幸福？
- 提醒您的朋友和家人您在戒烟。真正的朋友会为了您的健康而支持您的。
- 通过锻炼可以释放压力，去健身吧！您也许还有机会认识新朋友。
- 在戒烟过程中有没有奖励一下自己呢？用戒烟省下的钱去看场电影或者吃顿大餐吧。一天省下一包烟钱，一年省下的买烟钱都足够去旅游啦！
- 如果戒烟了，再吸一口，您就可能前功尽弃了！
- 恭喜您！戒烟成功了，再也不用为危害您健康的烟草买单了！
- 尽量远离那些让您联想到吸烟的人和地点，这有助于您戒烟，也能让您远离二手烟的侵害。
- 停止吸烟几周后，肺功能可以提升 30%！去骑自行车或散步吧，把您健康的肺好好利用起来。
- 您戒烟有一段时间了吧，参加一件有趣的活动激励自己吧。
- 很多烟民会为过去的吸烟行为感到悔恨。您为什么会感到悔恨呢？相信一定不是因为戒烟行动吧？
- 给所爱的人一个无烟之吻吧!他/她们会爱上您清新的口气并为您的戒烟行动感到高兴。
- 和支持您戒烟的人聊聊您戒烟的感受，您的感受是很重要的。
- 在戒烟的道路上您已经走了很远了，请继续保持！如果参加聚会、玩游戏、或者去餐馆，请记住不要吸烟。
- 吸烟会影响您的牙齿、牙龈和呼吸。戒烟能让您的牙齿更洁白、笑容更灿烂、口气更清新。

- 吸“淡口味”或“低焦油”卷烟并不能降低心脏疾病和肺部疾病的患病风险。所谓的“健康烟草”是不存在的。
- 尼古丁会让人上瘾。对于烟民来说，尼古丁会对您的生理和心理产生影响，使戒烟变得更难。
- 通过戒烟您省下了多少钱呢？计算一下吧，看看这些钱您可以用来买哪些别的东西？
- 现在保持无烟生活可能还是很难，但是您已经付出了很大的努力，您应该为自己感到骄傲。坚持住！
- 您的表现很好！戒烟可能很难，但是请保持自信。您一定可以做到的！
- 我们知道戒烟可能很难，但是它一定是值得的。请坚持无烟生活，您一定可以做到！
- 当您想吸烟的时候，试着拿一支笔、一根筷子、或一个吸管之类的东西，但是千万不要寻找烟!
- 在任何年龄戒烟均可获益。戒比不戒好，早戒比晚戒好，越早戒烟，健康获益越大。戒烟者可以比持续吸烟者更长寿。
- 您表现得很好！戒烟可能很难，但是请保持自信，您一定能做到！
- 我们知道戒烟可能很难，但是它一定是值得的。请坚持无烟生活，您一定可以做到！
- 压力或生气都可能是吸烟的诱因，试着开怀大笑，看看戏剧或搞笑电影。
- 婴儿和儿童吸入二手烟可能导致中耳炎、哮喘、肺功能下降，甚至突然死亡。
- 打开排风扇或在阳台吸烟都不能避免二手烟的危害，唯一能够有效避免二手烟危害的方法就是室内完全禁烟。
- 戒烟不是放弃而是收获！戒烟后肺部更干净，患心脏病的风险降低。
- 试着犒劳一下自己，给自己买件新衣服，或者是其他想买的东西，但是千万别吸烟！
- 戒烟还有助于增进骨和关节健康，降低骨折的风险。
- 即使吸一口烟也会损害您的 DNA，从而增加患癌症的风险。继续保持不吸烟的状态，我们知道这很难做到，但想想戒烟的好处和吸烟的坏处，相信您一定能做到。
- （12 周时）恭喜！您已经完成了 12 周的戒烟短信干预。请继续坚持不吸烟，您能做得到！我们知道这不容易，但是非常值得！现在开始，我们将减少短信量，继续追踪您未来 12 周的戒烟情况。

戒烟后的随访（13-24 周）

※说明：可根据具体情况调整短信内容的频率或只询问吸烟状态。

应对渴求

1. 特别想吸烟的时候，深呼吸、喝水、做点别的事情，告诉自己不要马上吸烟，再强的吸烟渴求也会在几分钟后慢慢消失。
2. 保持您周围有可以吃到的东西，试着吃个苹果、吃点葡萄干、瓜子或口香糖。
3. 特别想吸烟的时候，赶紧去洗下手。

4. 让自己忙碌起来，特别是让双手忙起来。
5. 告诉自己，一定不能让自己失望。
6. 给自己正能量，寻找健康的释放压力或宣泄情绪的方式。如果您正感到烦恼，深呼吸（用 10 秒左右的时间慢慢的深吸气，保持 1 秒，然后缓慢呼气）。
7. 想象一个舒缓、愉快的场面，例如在安静的小河边。不想别的，只想象一下您就身处这一安静舒适的环境中。
8. 如果实在忍不住，请点燃一根蜡烛或别的，千万不是香烟。
9. 到外面走走，或者到另外一个房间。试图改变您现在正在做的事情。
10. 散步或做点别的运动。
11. 喝瓶水，大量喝水有助于消除渴求。
12. 让双手忙碌起来，做点手工也不错。
13. 不妨打开手机玩个游戏。
14. 嚼口香糖。
15. 再做做深呼吸。
16. 给好朋友打个电话，或发条短信，特别是支持您戒烟的朋友。
17. 喝水、运动和放松是三种最有效的方法。
18. 洗个热水澡。热水澡会帮助您缓解吸烟渴求，洗完澡后刷刷牙，享受口腔新鲜而没有烟味的感觉，然后离开浴室，您的吸烟渴求将会消失。
19. 听听音乐，读读书可以让您放松。
20. 试图远离喝酒，因为喝酒可能引起吸烟的欲望。
21. 等待 15 分钟，可以拿本杂志读一读，听听歌，玩玩游戏。渴求几分钟后将渐渐消失。
22. 记住，每位戒烟者都要面对吸烟的渴求，渴求一般持续 5-10 分钟，虽然很难受，但是您一定能熬过，写下最容易让您分散渴求的事情，然后尝试去做。
23. 没有时间散步或跑步？那么花几分钟时间上下楼梯，即使这样也能增加您的能量，对抗吸烟渴求。
24. 算一下戒烟后您每个月节省了多少钱，一年节省了多少钱，计划一下如何花费这些钱。
25. 让嘴巴忙起来，吃点东西，比如口香糖、水果、零食等都可以。
26. 吸烟渴求很快就会消失，记住，一定不要吸烟，哪怕是吸一口，也可能使您再次回到吸烟状态。
27. 提醒自己戒烟的好处，并记录下来。
28. 锻炼一下身体，比如深呼吸、放松肌肉、瑜伽、按摩都可以。或者不妨读个笑话。
29. 记录或大声说出您戒烟的原因，这样可以抵制吸烟的渴求。
30. 记住，让自己忙点，做点事情比不做任何事情好。而且，特别想吸烟的感觉来的次数越多，离您彻底戒烟的日子也越近。

调整心情

1. 戒烟后，出现这些情况是正常的，每个人都会出现一些戒断症状。这是因为身体需要重新适应没有尼古丁的生活。戒烟后，您可能有强烈的吸烟欲望。
2. 好消息是，这些症状通常只持续几个星期，然后就会消失。坏消息是，对某些人来说，这些症状是如此强大和令人不愉快，以至于他们想重新开始吸烟以缓解这些症状。我们相信您一定能挺住！
3. 有没有其他方式可以缓解戒烟的不适呢？您可以尝试慢跑、散步、嚼口香糖、嗑瓜子等方法。
4. 不要让烟瘾挡住您戒烟的脚步。做一些运动，例如慢跑、散步、跳舞或者打太极拳等都可以帮助您有效地克服烟瘾。
5. 您准备越充分，戒烟就越顺利。把生活安排紧凑，让自己忙起来，您就不会想吸烟了。
6. 深呼吸！困难是暂时的，烟瘾也是暂时的。多和支持您戒烟的人在一起，这对您很有帮助。
7. 戒烟过程中多吃水果或蔬菜，进行体育锻炼，这将帮助您减轻压力，增加信心，也让您的生活更加美好。
8. 今天压力很大？千万不要依靠吸烟来缓解压力。出去活动活动、听听音乐、喝点水或者吃点东西吧。
9. 在戒烟期间每天喝大量的水，既可以保证您体内水分充足，也可以帮您抵抗烟瘾。
10. 让我们开始吧！薄荷糖、水和零食可以帮您克服一天的烟瘾。生活就是由选择构成的，戒烟是您做出的正确选择。
11. 我们知道戒烟十分困难，但是请坚持住！您一定可以成功！做一些能让您心情愉悦的事吧，只是不要吸烟。
12. 您可能不像刚开始戒烟时那么自信了，也许还会考虑放弃。不管怎样都要坚持住。为了健康，戒烟是能做到的！
13. 休息一下让自己放松。小睡一会儿、洗个澡、跑步或者大喊几声，这些活动可以帮您克服烟瘾。您一定可以成功！
14. 锻炼可以帮您减轻压力，从而降低您对吸烟的渴望。请每天抽出一点时间来锻炼身体吧。
15. 需要做件事情帮您转移注意力吗？约上朋友聊聊天或看看电影，享受点水果和小零食吧。
16. 您的业余时间要做点什么呢？在没有烟的环境里，读一本心爱的书或杂志，听一张新专辑或做一道新菜，是不是很幸福？
17. 提醒您的朋友和家人您正在戒烟。真正的朋友会为了您的健康而支持您的。
18. 回想并记录良好感受：例如节省下一大笔开销；口气变得清新；食物的滋味尝起来更鲜美；家人的关系更融洽；精力更加充沛，精神状态更加饱满。
19. 去刷个牙，口气清新，呼吸舒畅或许能改善您的心情。

20. 担心戒烟会令您发胖？锻炼！从运动中获得的内啡肽会改善您的心情，您会看起来感觉更好哦！
21. 感觉心情烦躁是正常的，某些日子会很难受。相信自己一定能挺过来！出去走走，玩一下，放松自己。
22. 您知道吗？压力过大是导致戒烟失败的最主要原因之一。休息 5 分钟再回来工作。
23. 我们知道现在烟的诱惑有多大，当您觉得想抽烟是正常的，不要因此对自己感到失望。集中精力做别的事情，您可以做到抵制诱惑！
24. 请一口烟都不要吸，即使吸一口烟也会大大增加重新吸烟的危险。虽然过程辛苦，但是非常值得。
25. 我们知道您现在一定觉得很难！请记住，一个无烟的生活意味着降低以后患肺癌、心脏病、牙齿发黄等的风险。
26. 压力太大让您觉得难受？重新回到吸烟生活不是您要的结果。给亲人或朋友打个电话，请他们帮助和支持您戒烟。
27. 什么事情让您高兴？音乐？慢跑？发短信给您最好的朋友？不管是什么事情，现在就做，但不要吸烟。明天您会很高兴。
28. 听听音乐，读读书可以让您放松一下，缓解紧张情绪。
29. 通过锻炼可以释放压力，去健身吧!您也许还有机会认识新朋友。
30. 在戒烟过程中感觉烦躁或脾气暴躁，都是正常表现，但是您知道这些是完全能够克服的，相信自己!

为什么戒烟

1. 《中国吸烟危害健康报告》指出，烟草烟雾中已发现数百种对人体有害的成分，其中包括近 70 种是明确的致癌物。
2. “我戒烟是因为我弟弟看不起我吸烟，我不希望以后他也吸烟”。
3. 促进健康，为了家人和朋友，可以到更多无烟场所。
4. 只要是烟就有害，没有“安全的卷烟”。所谓的“过滤嘴卷烟”、“低焦油卷烟”或“中草药卷烟”都不能降低吸烟带来的危害。
5. 《中国吸烟危害健康报告》指出，吸烟可导致肺癌、喉癌、肝癌、宫颈癌等多种癌症。
6. 吸烟可增加患肺结核的风险，而且不利于肺结核的治疗。
7. 吸烟会伤害血管，增加血液粘稠度，容易引起冠心病、中风等心脑血管疾病。
8. 吸烟会损害性功能，降低生育能力，导致男性阳痿、精子质量下降，影响女性受孕。
9. 准妈妈吸烟或吸入二手烟都会危害自己和宝宝的健康，容易发生宫外孕、流产、早产、先天畸形、婴儿死亡等危险。
10. 近年来糖尿病患者日益增多，吸烟也是导致糖尿病的重要原因，而且还会增加糖尿病血管并发症的风险。
11. 《中国吸烟危害健康报告》指出，中国每年因吸烟死亡的人数超过 100 万,高于结核、

艾滋病和疟疾导致的死亡人数之和。

12. 无论时间长短，只要吸入二手烟都会对健康产生危害。
13. 二手烟中有大量致癌物，即使室内通风换气也不能完全消除它的危害。
14. 戒烟后，由于吸烟造成的健康损害就会逐步得到改善。
15. 戒烟越早，对健康的益处越大。早戒比晚戒好，戒比不戒好。
16. 您每年花多少钱买烟？设想一下，戒烟可以省下多少钱？您可以用这些钱做哪些更有意义的事情？
17. 您吸烟也会导致您家人患上多种疾病，在吸烟前请考虑家人的健康。
18. 为了健康的呼吸、清新的口气、美丽的容颜，坚持戒烟吧!
19. 您知道吗？吸烟还可导致骨密度下降和骨折。
20. 戒烟以后的生活很美好，学会享受无烟的生活吧！
21. 停止吸烟几周后，肺功能可以提升 30%！
22. 戒烟可降低肺癌、冠心病、慢性阻塞性肺疾病等多种疾病的患病风险，也有利于疾病的康复。
23. 每年全球因为吸烟死亡的人将近 600 万人，平均每 6 秒就有一个人因为吸烟而死亡。
24. 婴儿和儿童吸入二手烟可导致中耳炎、哮喘、肺功能下降，甚至突然死亡。
25. 任何年龄戒烟均可获益。早戒比晚戒好，戒比不戒好，越早戒烟，健康获益越大。戒烟者可以比持续吸烟者更长寿。
26. 《中国吸烟危害健康报告》指出，与不吸烟者相比，吸烟者的平均寿命要缩短 10 年。
27. 您知道吗？吸烟还可损伤您的牙齿和眼睛，引起皮肤老化。
28. 吸入二手烟可导致肺癌、乳腺癌和冠心病等众多疾病。
29. 只要吸烟就会危害健康，即使偶尔少量吸烟也会对您的健康造成危害。
30. 吸烟会伤害您的肺，并增加肺部感染的风险，导致肺病。

© 2022 Liao Y. et al.
